# Detection of Drug-Drug Interactions by Modeling Interaction Profile Fingerprints

**DOI:** 10.1371/journal.pone.0058321

**Published:** 2013-03-08

**Authors:** Santiago Vilar, Eugenio Uriarte, Lourdes Santana, Nicholas P. Tatonetti, Carol Friedman

**Affiliations:** 1 Department of Biomedical Informatics, Columbia University Medical Center, New York, New York, United States of America; 2 Department of Organic Chemistry, Faculty of Pharmacy, University of Santiago de Compostela, Santiago de Compostela, Spain; University of Edinburgh, United Kingdom

## Abstract

Drug-drug interactions (DDIs) constitute an important problem in postmarketing pharmacovigilance and in the development of new drugs. The effectiveness or toxicity of a medication could be affected by the co-administration of other drugs that share pharmacokinetic or pharmacodynamic pathways. For this reason, a great effort is being made to develop new methodologies to detect and assess DDIs. In this article, we present a novel method based on drug interaction profile fingerprints (IPFs) with successful application to DDI detection. IPFs were generated based on the DrugBank database, which provided 9,454 well-established DDIs as a primary source of interaction data. The model uses IPFs to measure the similarity of pairs of drugs and generates new putative DDIs from the non-intersecting interactions of a pair. We described as part of our analysis the pharmacological and biological effects associated with the putative interactions; for example, the interaction between haloperidol and dicyclomine can cause increased risk of psychosis and tardive dyskinesia. First, we evaluated the method through hold-out validation and then by using four independent test sets that did not overlap with DrugBank. Precision for the test sets ranged from 0.4–0.5 with more than two fold enrichment factor enhancement. In conclusion, we demonstrated the usefulness of the method in pharmacovigilance as a DDI predictor, and created a dataset of potential DDIs, highlighting the etiology or pharmacological effect of the DDI, and providing an exploratory tool to facilitate decision support in DDI detection and patient safety.

## Introduction

Drug-drug interactions (DDIs) are a major cause of morbidity worldwide and a leading source of treatment inefficacy. For this reason, DDIs cause great concern in patient safety and pharmacovigilance. Adverse drug events (ADEs) may occur when drug combinations target shared metabolical and pharmacological pathways altering the efficacy and safety profile of the drugs. Potential DDIs are evaluated for experimental drugs pre-clinically during development and then monitored by drug safety surveillance programs after they enter the marketplace. The development of predictive tools to help study possible DDIs is of great interest to pharmaceutical companies and regulatory authorities, such as the United States Food and Drug Administration (FDA) [1]. These organizations are interested in better methods to detect and assess drug interactions [2].

Depending on the seriousness of the DDI, different measures are carried out ranging from the introduction of warnings in drug labels to the withdrawal of drugs from the market. As an example, in August 2008 the FDA [1] issued a warning about the possibility of developing rhabdomyolysis, a condition related to severe muscle injury, through combination treatment with simvastatin and amiodarone. In contrast, mibefradil, a calcium channel blocker approved by the FDA [1] in June 1997, was withdrawn from the market shortly after due to potential harmful interactions with drugs that prolong the QT interval [3].

In previous work, we proposed a method that used the DDI DrugBank database along with molecular similarity for detecting DDIs [4]. Medicinal chemistry researchers have exploited the concept of molecular similarity for years [5]–[12], where the basic idea is that ‘structurally similar molecules are likely to have similar biological properties’. Molecular fingerprints, digital representation of chemical features, are useful representations for comparing the structural similarity between compounds [10]–[13]. The basic idea in the development of a molecular fingerprint is to represent molecules through a vector that codifies in different positions the presence/absence of structural features. However, fingerprints could be designed to codify not only molecular structure information but also different biological properties.

**Figure 1:**
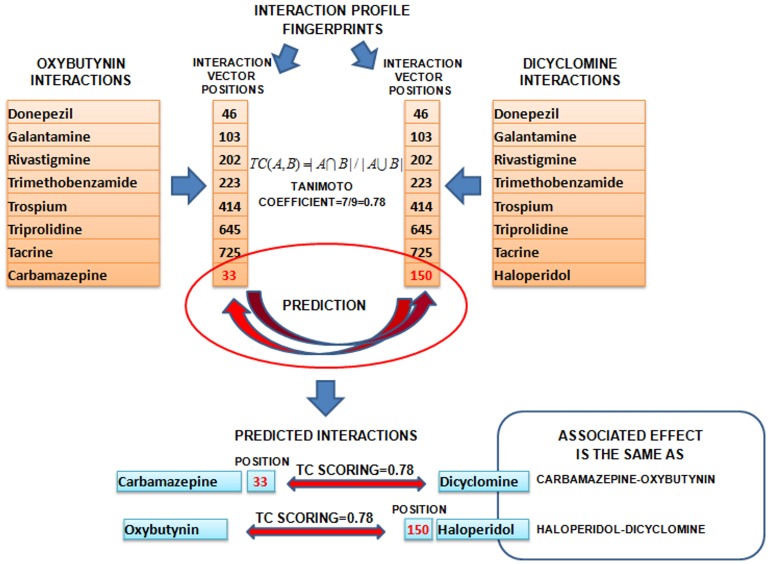
Examples of interaction profile fingerprints (IPFs) calculated for the drugs oxybutynin and dicyclomine. The similarity of both fingerprints is measured through the TC coefficient. The drugs corresponding to the non-intersecting interactions for the pair are assigned the TC score and form part of the prediction of the model. The effect associated by the interaction is the same as the original interaction source that generated the prediction.

Following the concept of predictive models based on adverse drug event profiles [14]–[15] and comparing drug pairs through molecular fingerprints [12], we developed a model to predict DDIs based on the comparison of, what we call, an interaction profile fingerprint (IPF). The IPF codifies the known interaction partners of a given drug as a binary vector of 1′s and 0′s. Two different interaction fingerprints can be compared using the Tanimoto coefficient (TC), a general method for comparing the similarity of two sets [16]. Our motivating hypothesis is as follows: if drug *i* and drug *j* are similar according to their interaction fingerprints, then drug *i* will interact with the same drugs as drug *j* with a probability related to the similarity of their fingerprints and vice versa. Figure 1 shows how the interactions of two drugs, oxybutynin and dicyclomine, are transformed into vectors, which are fingerprints, and then compared using the TC. The drugs associated with the non-intersecting interactions are predicted to participate in interactions with a probability proportional to the TC score (see Figure 1). For example, we predict carbamazepine interactions with dicyclomine with a probability proportional to 0.78 (Figure 1).

The model we developed combines the interaction profile similarity information using the DDIs specified in DrugBank to obtain new DDIs, but data from other sources could also be used. The model results were validated using Drugs.com [17] and Drugdex [18] databases as reference standards. We provided in the Table S1 of the Supporting Information a database with 17,230 DDI candidates predicted by the model along with the possible biological effects.

## Methods

### Generation of the Established Drug-drug Interaction (DDI) Database (Matrix M_1_)

We collected the database from DrugBank [19] in a previous publication [4]. Only small approved drugs, not including proteins and peptides, were introduced in the previous model resulting in DDI information for 928 drugs and a set of 9,454 unique DDIs. Although we used the same dataset in the current article, improvements through future updates in the DrugBank database or the use of other important sources of DDIs, such as Drugs.com database, could be beneficial. This step would require an overall recalculation of the interaction profiles.

We transformed the set of collected DDIs into a 928×928 binary matrix M_1_ with value of 1 representing an interaction between two drugs and value of 0 representing no interaction. The model included information about the pharmacological effect of the interaction associated with pairs of drugs as part of the process (e.g, the entry in DrugBank for the DDI between oxybutynin and triprolidine is: two anticholinergics may cause additive anticholinergic effects and enhance their adverse/toxic effects).

### Generation of the Interaction Profile Similarity Matrix M_2_

The interaction profile similarity matrix M_2_ is calculated in three steps:

#### Interaction profile fingerprints (IPFs) calculation

We represented all the drugs included in the study by IPFs. The concept of IPFs is similar to molecular structure fingerprints [16], [20]. The basic idea in IPFs is to represent the drug interactions for a particular drug as a vector codifying the presence of interactions in specific positions. As an example, in Figure 1 the interactions between oxybutynin and all other drugs are codified as different vector positions (33, 46, 103, 202, 223, 414, 645, 725). Only the positions whose value is 1 are stored in vector-position notations. This is a very efficient way to represent a sparse binary matrix. The same process is carried out for the drug dicyclomine that shares 7 out of 9 unique interactions with oxybutynin (46, 103, 150, 202, 223, 414, 645, 725). The transformation of the molecules into IPFs facilitates comparison.

#### Computation of similarity between fingerprints

We used the Tanimoto coefficient [16], also known as the Jaccard index, to compute similarities between all the IPFs. The TC between two fingerprints A and B is defined as the ratio between the number of features/interactions in the intersection to the union of both fingerprints:
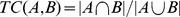



#### Construction of the matrix M_2_

We created a matrix so that the rows and columns represent drugs and each cell represents the interaction profile similarity based on the TC between the corresponding pair of drugs. We computed this matrix using the MOE software [21].

### Prediction of New DDIs (Matrix M_3_)

**Figure 2:**
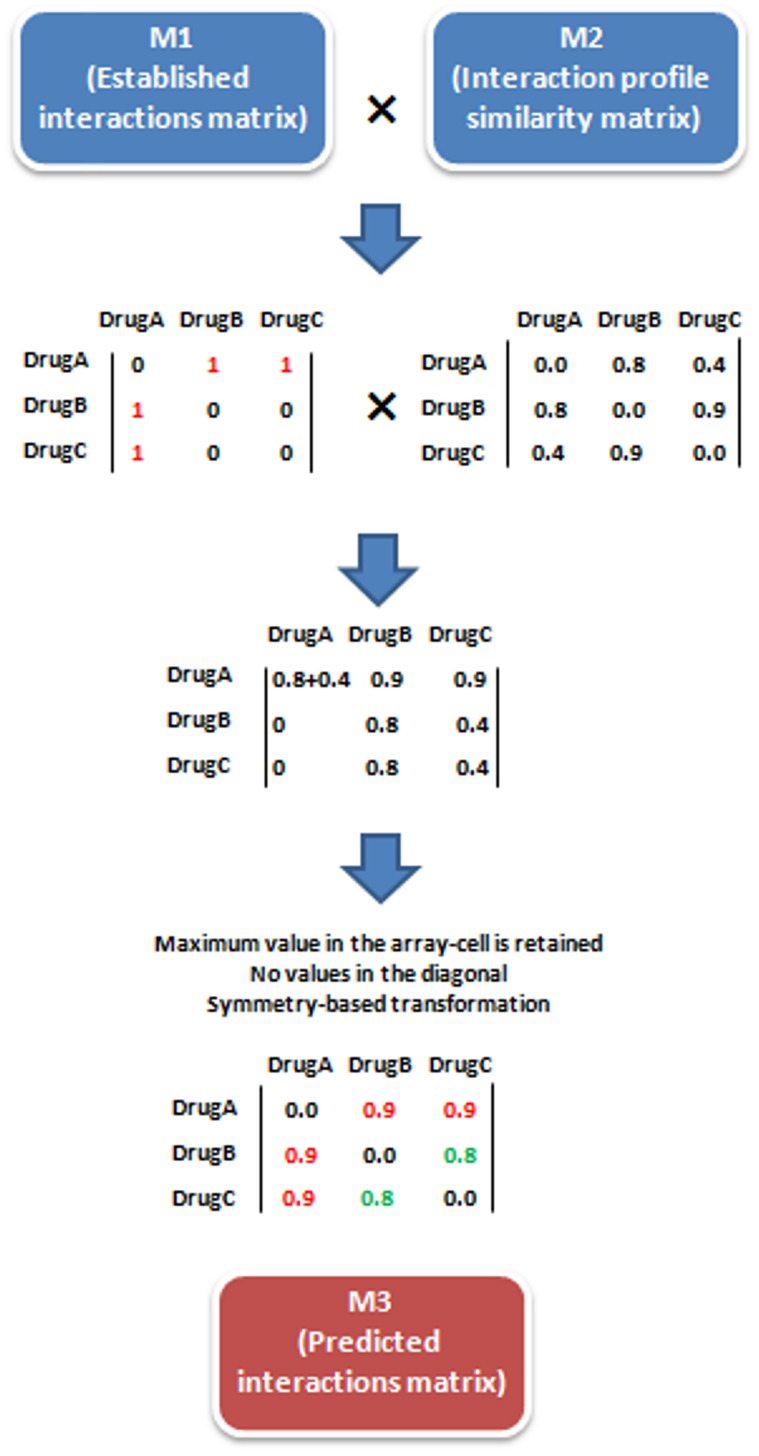
The model generates interactions through the multiplication of the matrix M_1_ (Established DDI matrix) by the matrix M_2_ (Interaction profile similarity matrix. Note that each cell shows the TC between drugs A, B and C but interactions with more drugs are considered to calculate the TC value). The values in the diagonal of the matrices are set 0 since drug interactions with themselves are not taken into account. In the final matrix M_3_ only the maximum value in the multiplication-array in each cell is preserved and a symmetry-based transformation is carried out retaining the highest TC value. In the example, the initial interactions A–B and A–C (red color) have a TC score of 0.9 in the matrix M_3_. The system generated a new predicted interaction between B and C with a TC score of 0.8 (green color).

To calculate the matrix M_3_ with new predicted interactions, we multiplied the matrix M_1_ (Established DDI database matrix) by the matrix M_2_ (Interaction profile similarity matrix) (see Figure 2). It is worth noting that the values in the diagonal of the matrices M_2_ and M_3_ are 0 since the interaction of a drug with itself is not taken into account. Although the model could generate multiple scores for the same interaction based on similarities from different pairs, we only considered the predicted interaction with the highest TC value. For this reason, in each cell of the product of the matrices, only the highest value in the array-multiplication is retained (see Figure 2). We transformed the resulting matrix into the symmetric matrix M_3_ considering the highest value (TC) for each pair of drugs. A set of new predicted DDIs are then generated from M_3,_ and the biological effect provided by the initial DDI source in M_1_ is captured and associated to the new DDIs. As an example, Figure 1 shows how we used a known interaction between haloperidol and dicyclomine to predict an interaction between haloperidol and oxybutynin. In addition, we assigned the biological effect of the known interaction “Increased risk of psychosis and tardive dyskinesia” to the predicted interaction.

### Evaluation

#### Hold-out validation

We divided the database randomly in two sets: training and test sets. We performed two evaluations by moving 15% and 30% of the initial interactions to the test set, and by constructing the model with the remaining interactions in the new matrices M_1_ and M_2_. To evaluate the performance in the training and test sets, we plotted the Receiver Operating Characteristic (ROC) curves and used the area under this curve (AUROC) as a summary statistic.

#### Test evaluation

For the assessment of the performance of the model we used four different independent test sets, which do not contain any interactions from the initial DDI database M_1_: A) the top 100 DDIs generated by the model according to the TC value, B) a random set of 100 drug interactions with a TC≥0.7, C) a random set of 100 drug interactions with a TC≥0.4, and D) the interactions generated by the model with a TC≥0.4 for the 50 most frequently sold drugs in 2010 [22]. We used the Interaction Checker from Drugs.com [17] and Drugdex (Micromedex) database [18] as a reference standard to determine the number of interactions that were correctly predicted. The level of documentation in the reference standard ranges from ‘interactions clearly established through controlled studies’ to ‘limited studies but the interactions are recognized through pharmacological knowledge’. We calculated precision and enrichment factor compared to random selection (see formulas in Table 1) for the four sets as measurements of the performance. In addition, in order to provide more information, we plotted a Receiver Operating Characteristic (ROC) curve for test set D. The predicted biological/pharmacological effect associated with the DDIs was also assessed based on the Drugs.com and Drugdex databases.

**Table 1:** Model performance in the four independent test sets A, B, C and D along with random results.

Test set model performance				
Set A: TOP 100 predicted interactions according to TC value				
TP	FP	Precision	EF	*p*-value
50	50	0.50	2.63	<.001
Set B: 100 predicted interactions randomly selected with TC≥0.7				
TP	FP	Precision	EF	*p*-value
43	57	0.43	2.26	<.001
Set C: 100 predicted interactions randomly selected with TC≥0.4				
TP	FP	Precision	EF	*p*-value
45	55	0.45	2.37	<.001
Set D: Predicted interactions with TC≥0.4 for the TOP 50 drugs sold in 2010				
TP	FP	Precision	EF	*p*-value
640	744	0.46	2.43	<.001
Random system calculated for the TOP 50 drugs sold in 2010				
TP	FP	Precision	–	–
19	81	0.19		

TP = True positives, FP = False positives, Precision = TP/(TP+FP),

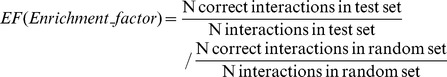

#### Random results evaluation

The results obtained by the model were compared to random expectations. We created a random system taking into account the list of 50 most frequently sold drugs in 2010. The top 50 list included 50 generic drug names but we only included 41 generic names in the system. Nine of these drugs are not represented in the DrugBank DDI database. We cannot generate interaction predictions for these drugs so we removed them from consideration. These drugs were mometasone, ezetimibe, ferrous fumarate, naloxone, sitagliptin, latanoprost, insulin glargine, insulin aspart, and omega-3-acid ethyl esters.

The number of possible interactions for 41 drugs in a matrix of 928 drugs is 37,187 (927 factorial 41 times). We estimated the number of positive cases as 7,068 interactions found in Drugs.com and/or Drugdex and used a one-sided Fisher’s exact test to calculate significance (p-value).

## Results

We combined similarity information by means of interaction profile fingerprint-based modeling with the initial database containing 928 drugs and 9,454 DDIs, as described in the Methods section. The final model generated a matrix of 430,128 DDI scores. Among these interactions are the initial 9,454 DrugBank DDIs used to develop the model. We evaluated the performance of the model through hold-out validation and external test series.

### Hold-out Validation Model Performance

**Figure 3:**
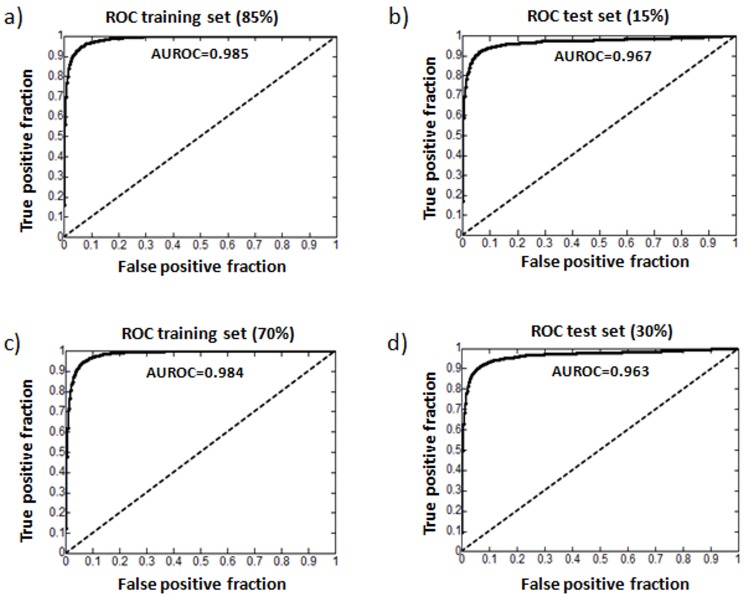
ROC curves in the hold-out validation process: a) training set with the 85% of the DrugBank interactions; b) test set with the 15% of the extracted DrugBank interactions; c) training set with the 70% of the DrugBank interactions; d) test set with the 30% of the extracted DrugBank interactions.

We performed two different evaluations by dividing the initial database into training and testing subsets. In the first we moved 15% of the interactions from the training to the testing set and in the second we moved 30%. Using DrugBank DDIs as true positives we plotted ROC curves and computed the area under the curve (AUROC). We found an AUROC = 0.967 for the 15% hold-out and AUROC = 0.963 for the 30% hold-out (Figure 3). The stability of the model is barely affected even when we removed twice as many interactions. However, high performance in these sets is expected since the similarity matrix was generated using drug interaction profile information where the drugs and interactions in DrugBank have been specified and form a closed system. As an example, the interactions retrieved for two drugs that share all the interactions in the initial database have the maximum score (TC = 1). For this reason, further evaluation of the model using independent test sets with no interactions previously collected in the initial DDI database is a necessary step to prove the prediction power.

### Test Set Model Performance

#### Test set A. The TOP 100 drug interactions predicted by the model (TC_threshold = 0.92)

We found 50 out of 100 interactions in Drugs.com and/or Drugdex (Micromedex) databases. The precision of this external set is 0.50. Random expectations (see Methods section) selecting 100 interactions in a set of 37,187 possible interactions where there are 7,068 positive cases, would detect 19 positive cases (random precision = 0.19). The performance of the model showed a 2.6 fold (p<0.001) enrichment factor (see Table 1 and Table S2).

#### Test set B. Random set of predicted 100 drug interactions with a TC≥0.7

We found similar results for the second independent test where 43 out of 100 random interactions with a TC≥0.7 were in the reference standard. The precision in the second test set is 0.43 and the enrichment factor 2.3 (p<0.001) (see Table 1 and Table S3).

#### Test set C. Random set of predicted 100 drug interactions with a TC≥0.4

In the evaluation of the third test, we detected in our reference standard 45 out of 100 random interactions with a TC≥0.4 (see Table 1 and Table S4 for more details).

#### Test set D. Interactions predicted for the 50 most frequently sold drugs in 2010

**Figure 4:**
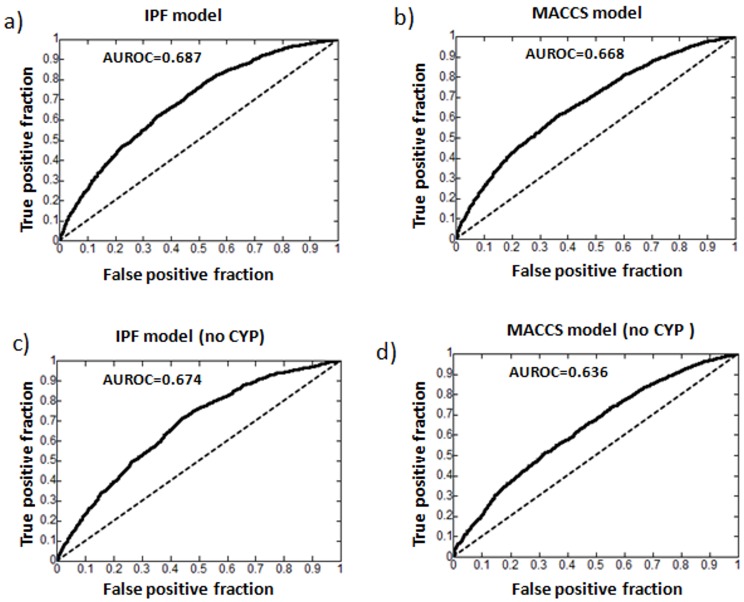
ROC curves for test set D: a) ROC curve generated by the IPF model for test set D. Interactions for the top 50 drugs (41 generic names) confirmed in drugs.com/drugdex were considered as true positives within all the possible interactions in a matrix of 41×928 drugs. Interactions already in the initial DrugBank DDI database (matrix M_1_) were not included in the analysis; b) ROC showed by a model applied to test D using MACCS fingerprints; c) ROC curve calculated by the IPF model for test set D but excluding CYP interactions; d) ROC showed by the MACCS fingerprints model applied to the test D without CYP interactions.

**Figure 5:**
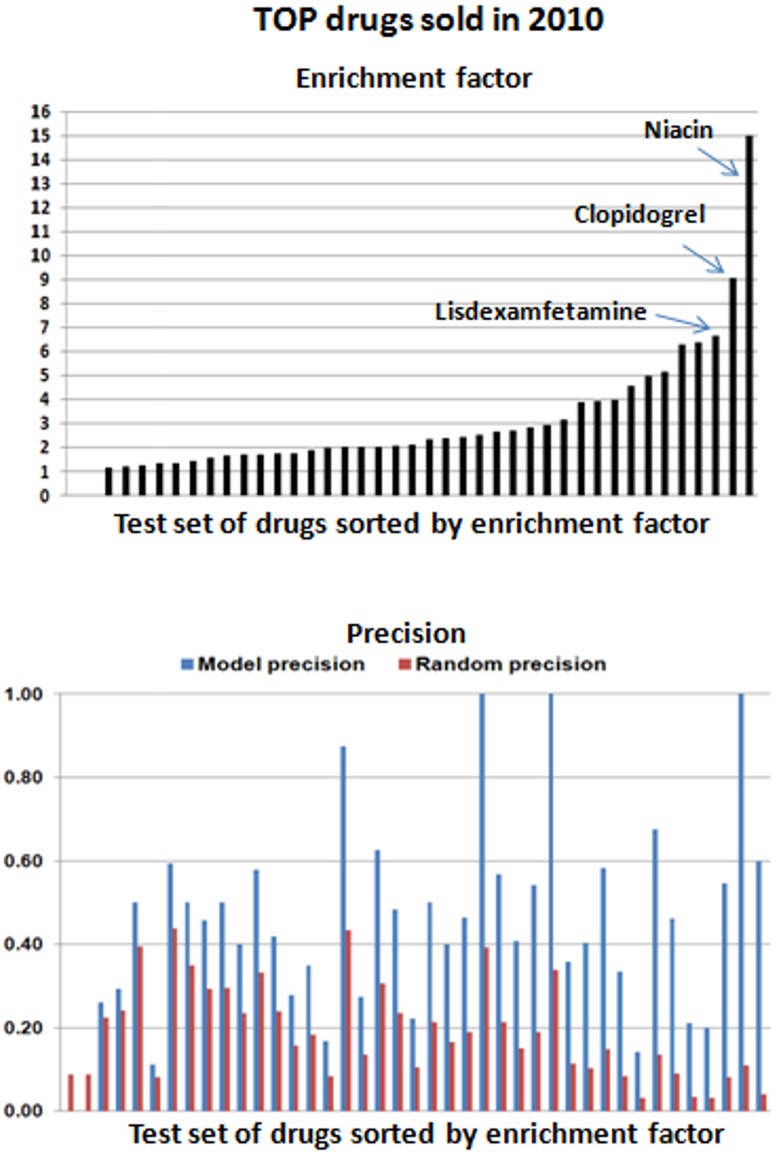
Enrichment factor (a) and precision (b) achieved by the model regarding random results for top drugs sold in 2010 (test set D). The test set of drugs are sorted according to the enrichment factor.

46% of the generated interactions with TC≥0.4 were confirmed in the reference standard. The model presents an enrichment factor of 2.4 (p<0.001) (see Table 1 and Table S5 for a detailed description of the evaluation). In addition, we plotted the ROC curve taking into account as true positives all the interactions in the set confirmed in drugs.com/drugdex (see Figure 4a). The area under the curve is 0.69. Figure 5 shows the enrichment factor and precision achieved by the model for each drug. Out of the 50 drugs, we included 41 in the evaluation. Nine drugs were not taken into account because they were not included in our initial DrugBank DDI database and the model could not predict any interaction.

Our method outperforms other commonly used approaches. A method recently published by our research group based on molecular structure similarity [4] showed less predictive capacity (AUROC = 0.668) compared to our model (AUROC = 0.687) when applied to the test set D (see Figure 4). In addition we tested if our model could predict pharmacodynamic interactions as well as pharmacokinetic. Using DrugBank annotations, we identified and removed any interactions between drugs with shared metabolism by a cytochrome p450 (CYP) metabolizing enzyme (1A2, 2B6, 2C8, 2C9, 2C19, 2D6, 2E1, 3A4, 3A5 and 3A7) [19]. 14,242 interactions in the test set D included in the CYP list were removed. We found that our approach performed nearly as well (AUROC = 0.674), but that the performance of the molecular structure based approach performed was reduced by 3% (AUROC = 0.636) (Figure 4).

### Pharmacological Effect Prediction

A pharmacology expert manually reviewed and compared the pharmacological effect described in the predicted interactions for the test sets to the effect found in Drugs.com and Drugdex databases. The interactions predicted by the model belong to two categories: some are generated comparing interaction profiles of pairs of drugs in the same pharmacological class, whereas the origin of other interactions resides in the comparison of the profile fingerprints of pairs of drugs that are not in the same class.

For the test set A with the top 100 interactions, 43 out of 50 true interactions (86%) were confirmed to have the same effect as the described in our reference standard (see Table S2). We found a similar result for the test set B (100 interactions with TC≥0.7) where the effect in 36 out of 43 confirmed interactions (84%) was considered correct (see Table S3 for a detailed description). For these test sets, the model generated the majority of the reviewed interactions through the comparison of pairs of drugs catalogued in the same or similar pharmacological class (48 out of 50 and 38 out of 43 for test A and B respectively). As the TC values decrease so does our confidence in the predicted effect as these predictions result from comparing pairs of drugs with different pharmacological profiles. In test set C with TC≥0.4, the pharmacological effect was correct for the 66.7% of the interactions, i.e. 30 out of the 45 interactions found in the reference standard (see Table S4). For the last test set D, we carried out a more challenging evaluation and only the effect of the interactions generated through the comparison of pairs of drugs belonging to different pharmacological classes was evaluated. Out of the 640 correct DDIs predicted by the model for test set D, 215 were from comparing drugs belonging to different pharmacological classes. We reviewed the pharmacological effect for this set of 215 predicted interactions showing a percentage of correct classification of 59% (the effect was correct in 126 out of 215 cases).

Table 2 describes some example predictions from the test dataset D (detailed description is provided in Table S6) for which the model correctly detected interactions comparing drugs of different pharmacological classes as well as the effect produced by these interactions. For instance, the model detected that amoxapine, a tetracyclic antidepressant of the dibenzoxazepine family, has some similarity with the interaction profile of the antibiotic linezolid (TC = 0.40), and for this reason the model predicted the interaction escitalopram-amoxapine with a possible serotoninergic syndrome.

**Table 2:** Some examples of correct interactions predicted for the 50 most frequently sold drugs in 2010 in which the model generated interactions through the comparison of drugs belonging to different pharmacological classes.

Similar drug to A^1^	Predicted interaction DrugA-DrugB	Similar drug to B^1^	TC	Predicted effect
	Aripiprazole-Nelfinavir	Itraconazole	0.55	Increased effect of aripiprazole
	Aripiprazole-Atazanavir	Ketoconazole	0.45	Increased effect of aripiprazole
Alprazolam	Atorvastatin-Digoxin		0.40	Increased effect of digoxin
Midazolam	Atorvastatin-Omeprazole		0.51	Increased effect of atorvastatin
	Atorvastatin-Miconazole	Imatinib	0.43	Increased effect and toxicity of atorvastatin
	Buprenorphine-Trospium	Triprolidine	0.43	Possible increase adverse/toxic effects due to additivity
	Buprenorphine-Trimethobenzamide	Triprolidine	0.40	Possible increase adverse/toxic effects due to additivity
Felodipine	Conjugated_Estrogens-Oxcarbazepine		0.51	Decreased levels of estrogens
Gefitinib	Conjugated_Estrogens-Clarithromycin		0.47	Increased levels/toxicity of estrogens
Nifedipine	Conjugated_Estrogens-Cimetidine		0.40	Increased the effect of estrogens
	Duloxetine-Tolterodine	Tamsulosin	0.59	Possible decreased metabolism and clearance of Tolterodine. Changes in therapeutic/adverse effects of Tolterodine
	Duloxetine-Trimethobenzamide	Triprolidine	0.40	Possible increase adverse/toxic effects due to additivity
	Duloxetine-Sibutramine	Zolmitriptan	0.53	Increased risk of serotonin syndrome
	Escitalopram-Amoxapine	Linezolid	0.40	Possible serotoninergic syndrome
	Eszopiclone-Trimethobenzamide	Triprolidine	0.40	Possible increased adverse/toxic effects due to additivity
	Ethinyl_Estradiol-Trimipramine	Tacrolimus	0.43	Possible increased blood concentration of Trimipramine
Cisapride	Levofloxacin-Propafenone		0.44	Increased risk of cardiotoxicity and arrhytmias
	Methylphenidate-Linezolid	Rasagiline	0.52	Possible hypertensive crisis with this combination
Gefitinib	Norethindrone-Voriconazole		0.48	Possible increased serum concentration of norethindrone. Changes in the therapeutic and adverse effects
	Oxycodone-Trospium	Triprolidine	0.43	Possible increased adverse/toxic effects due to additivity
	Pioglitazone-Nelfinavir	Ketoconazole	0.51	Increased the effect of pioglitazone
Dihydroergotoxine	Salmeterol-Delavirdine		0.48	Increase salmeterol toxicity
Lidocaine	Salmeterol-Atazanavir		0.56	Increased risk of cardiotoxicity and arrhythmias
	Sildenafil-Clonidine	Terazosin	0.47	Increased risk of hypotension
	Simvastatin-Conivaptan	Imatinib	0.43	Increased effect and toxicity of statin
Bromazepam	Tadalafil-Rifabutin		0.50	Possible decreased serum concentration of Tadalafil. Changes in the therapeutic and adverse effects
Tadalafil	Zolpidem-Doxazosin		0.55	Risk of significant hypotension with this association

TC is the Tanimoto coefficient.

1 The similarity between drugs is based on the drug-drug interaction profile.

The model also predicted that levofloxacin could interact with propafenone, fluconazole, ibutilide, ranolazine, saquinavir and telithromycin with risk of cardiotoxicity and arrhythmias (see Table 2 and S6). The interactions were corroborated in Drugs.com database with a similar effect. We predicted other combinations, such as atazanavir-salmeterol, to cause cardiotoxicity and arrhythmias.

The model predicted possible hypertensive crisis with the combination methylphenidate and linezolid. The system generated the interaction because linezolid has a similar interaction profile as the monoamine oxidase inhibitor rasagiline (TC = 0.52) and the interaction methylphenidate-rasagiline was included in the initial database.

Among other examples, we also detected that the antidiabetic pioglitazone could interact with the macrolide antibiotic clarithromycin, and with the anti-HIV drugs indinavir and nelfinavir producing and increased effect of pioglitazone (see Table 2 and S6).

Although all the new DDIs generated by the model have corresponding predicted biological effects, it is important to take into account that as the TC value associated with the new interaction decreases so does the certainty of the associated effect.

## Discussion

The desirable and undesirable drug effects in patients are highly dependent on pharmacokinetic properties, such as absorption, distribution, metabolism and excretion (ADME), and pharmacodynamic properties, such as interaction with pharmacological targets. These important processes can be altered by the co-administration of different drugs at the same time. For this reason, drug interactions are an important problem in the surveillance of approved drugs and in the evaluation and development of new drug candidates. The FDA has shown its concerns to address this issue, and provides guidance to perform *in vitro* and *in vivo* drug interactions studies during the developmental stage of new drugs [2], [23]–[24].

A great effort has been made to develop *in silico* approaches, focused on the integration of *in vitro* data, to predict *in vivo* drug interactions [23], [25]. These models principally focused on metabolic interactions related to CYP enzymes. Other types of computational models to predict affinity for CYP enzymes based on molecular descriptors have also been developed [26]. Although many interactions are produced by the inhibition of metabolizing enzymes, there are also other possible mechanisms, such as interactions with transporters or pharmacological targets. Systems to further analyze pharmacodynamics interactions *in vivo* have been also described [27]. Other approaches to predict different types of DDIs have been recently published [28]–[29]; some of them take into account algorithms to detect interactions in adverse event reports [30], or text mining methods [31]. Our group has also recently described a large-scale DDI predictor based on molecular structure similarity to drug pairs [4]. Gottlieb *et al.* [32] have recently published a similar interesting large-scale approach to predict pharmacokinetic and pharmacodynamic DDIs. The authors used the concept of similarity to drug pairs, including different measurements, such as chemical structure, drug targets and side effect similarities, to infer new DDIs in a complex system with excellent results.

In this article, we developed a novel drug fingerprint based on drug interactions profile with successful application to DDI prediction and pharmacovigilance. Through the inclusion of interaction profile fingerprint-based similarity to the initial well-established DDI database, we constructed a large-scale drug interaction predictor taking into account different pharmacological effects caused by pharmacokinetic and pharmacodynamic characteristics of the drugs implicated in the interaction. The model generated some predicted interactions comparing the interaction profiles of pairs of drugs in the same pharmacological class, whereas a more challenging task is carried out when the interactions are generated comparing drugs belonging to different classes. The dataset of DDI candidates is available in the Table S1 of the Supporting Information for further study.

The aim of the model is to detect interactions when two drugs are implicated and does not account for co-DDIs or secondary interactions due to primary interactions. The development of a more complex and challenging model would be necessary to address this issue. Information about concentration of the drugs and environmental variables are not included in the model either. However, implicit bioavailability information has been incorporated since our initial DDI database contains examples where two drugs share the same metabolizing enzymes causing a higher bioavailable doses for one of the drugs implicated in the interaction.

Targets and drug promiscuity data was not directly introduced although implicit target information is taken into account since pharmacodynamic interactions were included in the system. As an example, Figure 4c has shown that model performance was not affected after eliminating possible CYP-related DDIs. Nevertheless, enhancement in our DDI system could also be achieved through the integration of metabolizing, transporters and pharmacological targets information provided by chemical databases such as PubChem [33]. Pharmacovigilance databases, such as the FDA’s Adverse Event Reporting System (AERS) [34], or the use of clinical data in Electronic Health Records (EHR) [35] could be also combined to further study possible DDI candidates.

**Figure 6:**
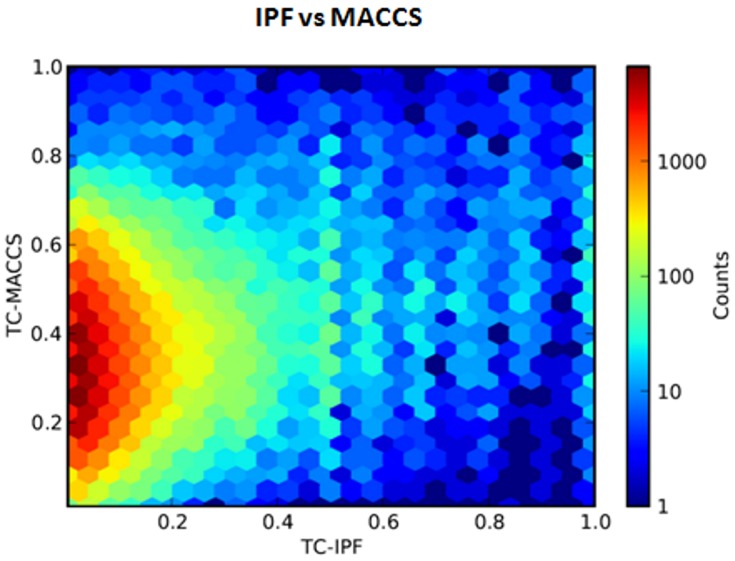
Comparison between the TC for all the pairs of drugs in a matrix of 928×928 using MACCS and IPF fingerprints. The correlation coefficient (r) calculated through linear regression is 0.167 and p<.0001.

Other types of models introducing 2D or 3D molecular structure data could be integrated in our system. The information provided by molecular structure can be different or complementary to IPF fingerprint data. We computed the correlation coefficient between the TC for all the pairs of drugs in the study using IPF and MACCS fingerprints. The results showed a low correlation coefficient of 0.167 (see Figure 6). However, it is noteworthy that there actually is a relationship between molecular structure similarity and interaction profile information in that if two drugs share similar interactions it is likely that they have some structural similarity. It is interesting to note that in the matrix of 928×928 drugs, 2,334 unique pairs were computed with a TC>0.70 using the structural fingerprints MACCS [36]. Using our IPFs, 3,332 pairs of drugs were established to have a TC>0.4. The comparison of both subsets showed 694 pairs of drugs in common whereas a random measurement would have yielded 18 common cases ( = 3332×2334/430128). When the structure of two molecules is compared through classical structural molecular fingerprints, the TC values are in the range of 0.85 for similar molecules. However, the TC range for establishing whether two molecules are similar is highly dependent on the molecular property information included in the fingerprint. In this article, TC values of 0.4 still indicate some level of similarity between two drugs related to interactions, as we show through the evaluation of the test sets C and D.

The model used only the DDIs described in DrugBank to generate new predictions. However, we evaluated the model using a larger set of interactions, such as those in Drugs.com and Drugdex, which contain many DDIs not described in our initial DDI database. The limitation in the data used to construct the model is likely to have influenced the results when using large DDI databases as a reference standard because DrugBank is a more limited resource of interactions. This could be an important reason why there are many cases where the model does not detect the DDIs described in the reference standard, resulting in false negative results. For instance, in the evaluation of test D, the model generated 71 possible interactions with a TC≥0.4 for the three HMG-CoA reductase inhibitors: atorvastatin, rosuvastatin and simvastatin. However, we found a total of 345 interactions containing these three drugs in Drugs.com/Drugdex databases that involved drugs included in our initial DrugBank database. This fact shows that there are many interactions undetected by our model when using a TC cutoff of 0.4. Lowering the TC cutoff will increase the sensitivity of the model but at the same time the false positive rate will be increased. Improvements in the system could be made by supplementing the DDIs and drugs in DrugBank with other sources of drugs and DDI information.

In our evaluation, false positives were deemed to be those that were not present in the reference standard. However, it is possible that some of these interactions have not yet been discovered or that some were not in the reference standard but could have been found if we used other sources of interactions as a reference standard. Furthermore, we further studied the false positive DDIs detected by our method in test set A using the INDI predictor [32] that provides a large scale state of the art method to predict pharmacokinetic and pharmacodynamic DDIs. 17 out of 49 DDIs were also candidates predicted by INDI suggesting agreement between both systems.

### Conclusion

In this article, we have designed a novel molecular fingerprint based on DDI profiles and developed a useful *in silico* model to predict new drug interactions and to specify their possible effects. The methodology, which can be applied on a large scale, was systematically validated through the evaluation of independent and external test sets and showed precision values ranging from 0.4–0.5 with more than two fold enrichment factor enhancement compared to random expectations. Through this DDI predictor, a database with 17,230 drug-drug interaction candidates along with the possible pharmacological effects is provided in the Supporting Information. This database can be combined with other exploratory tools, such as pharmacovigilance data analysis, to facilitate decision support in DDI detection.

## Supporting Information

Table S1 Predicted interactions (not included in the DrugBank DDI dataset) with a Tanimoto coefficient (TC) score ≥ 0.4 generated by the model.
(XLSX)

Table S2 Evaluation of the top 100 interactions (test set A).
(XLSX)

Table S3 Evaluation of a random set of 100 interactions with TC ≥ 0.7 (test set B).
(XLSX)

Table S4 Evaluation of a random set of 100 interactions with TC ≥ 0.4 (test set C).
(XLSX)

Table S5 Interactions predicted by the model with TC ≥ 0.4 and corroborated in Drugdex and/or Drugs.com databases for the 50 most frequently drugs sold in 2010 (test set D).
(XLSX)

Table S6 Evaluation of the pharmacological effect predicted by the model for the interactions generated with Tanimoto coefficient ≥0.4 for the 50 most frequently sold drugs by unit in 2010.
(XLSX)
